# Gene flow networks among American *Aedes aegypti* populations

**DOI:** 10.1111/j.1752-4571.2012.00244.x

**Published:** 2012-11

**Authors:** Anders Gonçalves da Silva, Ivana C L Cunha, Walter S Santos, Sérgio L B Luz, Paulo E M Ribolla, Fernando Abad-Franch

**Affiliations:** 1 Commonwealth Scientific and Industrial Research Organisation (CSIRO), Division of Marine and Atmospheric ResearchHobart, TAS, Australia; 2 Instituto Leônidas e Maria Deane (ILMD) – Fiocruz AmazôniaManaus, Amazonas, Brazil; 3 Universidade Estadual Paulista (UNESP)Botucatu, São Paulo, Brazil

**Keywords:** Amazonia, coalescent, dengue, disease spread, Migrate-N, ND4, networks

## Abstract

The mosquito *Aedes aegypti*, the dengue virus vector, has spread throughout the tropics in historical times. While this suggests man-mediated dispersal, estimating contemporary connectivity among populations has remained elusive. Here, we use a large mtDNA dataset and a Bayesian coalescent framework to test a set of hypotheses about gene flow among American *Ae. aegypti* populations. We assessed gene flow patterns at the continental and subregional (Amazon basin) scales. For the Americas, our data favor a stepping-stone model in which gene flow is higher among adjacent populations but in which, at the same time, North American and southeastern Brazilian populations are directly connected, likely via sea trade. Within Amazonia, the model with highest support suggests extensive gene flow among major cities; Manaus, located at the center of the subregional transport network, emerges as a potentially important connecting hub. Our results suggest substantial connectivity across *Ae. aegypti* populations in the Americas. As long-distance active dispersal has not been observed in this species, our data support man-mediated dispersal as a major determinant of the genetic structure of American *Ae. aegypti* populations. The inferred topology of interpopulation connectivity can inform network models of *Ae. aegypti* and dengue spread.

## Introduction

A network of nodes connected by edges provides a powerful framework to model connectivity in epidemiology (Proulx et al. 2005). Network topology, specified by the connections among nodes, is used to represent host or pathogen dispersal pathways at various scales, from contacts between individuals (Eubank et al. 2004) to links between cities along transportation networks (Grais et al. 2003; Colizza et al. 2006), and provides an effective framework to test different disease control strategies (Eubank et al. 2004). Hence, empirically determining network topology is a key component of efforts aimed at enhancing infectious disease control strategies.

In vector-borne diseases, such as dengue fever, analysis of disease spread at broad spatial scales (e.g., across cities, countries, or continents) requires knowledge about potential vector movement pathways (Takahashi 2004). Takahashi (2004) showed through modeling that intercity dispersal of *Aedes aegypti* can significantly influence the spatial dynamics of dengue. These models, however, made a number of simplifying assumptions, such as symmetrical movement between cities and a stepping-stone connectivity structure among cities. Examining the validity of these assumptions by testing hypotheses about vector movement at appropriate scales would increase the model’s resolution and utility.

Two main approaches have been used to study *Ae. aegypti* dispersal: mark–recapture (Trpis and Hausermann 1986) and population genetic analysis (Urdaneta-Marquez and Failloux 2011). Mark–recapture studies report female dispersal distances >500 m only when there is a lack of local oviposition sites (Trpis and Hausermann 1986; Harrington et al. 2005; Reiter 2007). Such small distances are relevant at the single-site scale, but do not assist in understanding movement patterns at broader national, regional, or continental scales. Limited dispersal implies that strong genetic structure should exist among populations (Wright 1943). Most studies, however, have rejected this hypothesis (Gorrochotegui-Escalante et al. 2002; Bosio et al. 2005; Urdaneta-Marquez et al. 2008; Hlaing et al. 2010), describing instead a paradoxical pattern of high levels of genetic differentiation within and low differentiation among urban sites. This observation has led to the proposal of man-mediated passive dispersal of eggs, larvae, and adults artificially increasing gene flow at broad spatial scales (Huber 2004). This hypothesis predicts that genetic similarity should be correlated with the rate of transportation, in particular commercial traffic, between localities. Thus, *Ae. aegypti* interpopulation differentiation would be lower among large, commercially important cities than among smaller rural towns, and a pattern of isolation-by-distance would be expected between rural towns and the urban centers with which they trade, reflecting some intermediate, restricted level of traffic.

Support for this hypothesis is compelling, with genetic studies across the species distribution describing the expected pattern (Gorrochotegui-Escalante et al. 2000, 2002; Huber 2004; Bosio et al. 2005; Merrill et al. 2005; Dueñas et al. 2009; Sukonthabhirom et al. 2009; Hlaing et al. 2010). However, Bosio et al. (2005) cautioned that multiple processes could generate similar allele frequency distributions (Holsinger and Weir 2009). Thus, genetic drift, natural selection, and common ancestry are also plausible mechanisms to account for the observed pattern of population differentiation. In addition, most studies to date have relied on estimates of gene flow (*Nm*) derived from fixation indices (*F*_ST_) through Wright’s equation, which are not robust to violations of the assumptions underlying the island model of population genetic structure (Whitlock and McCauley 1999). Thus, published information on *Ae. aegypti* dispersal do not provide adequate data to inform a network model such as that described by Takahashi (2004).

Yet, genetic data can shed light on the problem if analyzed within a coalescent framework. The coalescent model can ultimately tease apart different deterministic and stochastic effects by accounting for genealogical differences across loci and estimate relevant parameters, such as gene flow, for specific loci (Rosenberg and Nordborg 2002). While coalescent models are more powerful when used with multiple loci, one locus is often sufficient to test simple gene flow network hypotheses (Beerli and Palczewski 2010), which can be useful as a first step to inform epidemiological network models.

**Figure 1:**
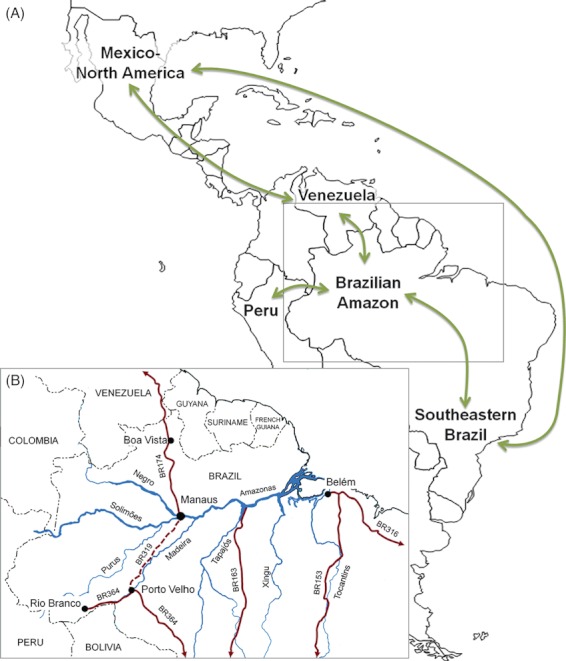
Geographic location of *Aedes aegypti* populations included in the study: (A) in the Americas (*N* = 2811 specimens from five locations: Mexico-North America, Venezuela, Peru, Brazilian Amazon, and southeastern Brazil); and (B) within the Amazon region (*N* = 74 specimens from four locations: Boa Vista, Manaus, Belém, and Rio Branco-Porto Velho). In (A), the model ranked first at the continental scale is represented by the arrows. In (B), major navigable rivers (Negro, Solimões, Purus, Madeira, Tapajós, Xingu, Tocantins, and Amazonas) and highways (BR174, BR319, BR364, BR163, BR153, and BR316) are presented; the broken line for BR319 indicates that this highway is only in use during a few months in the dry season and is therefore not used for routine commercial transport.

**Figure 2:**
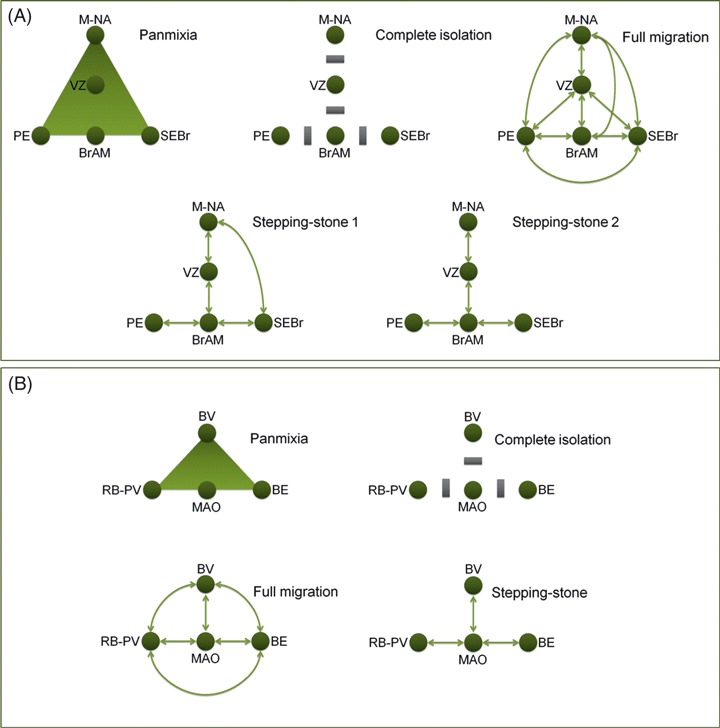
Models examined at each sampling scale: (A) continent; and (B) Amazon. BrAM, Brazilian Amazon; SEBr, southeastern Brazil; PE, Peru; VZ, Venezuela; M-NA, Mexico-North America; MAO, Manaus; BEL, Belém; BV, Boa Vista RB-PV, Rio Branco-Porto Velho. Arrows indicate gene flow between nodes.

Here, we use a Bayesian coalescent framework to test the support for alternative gene flow network models among American *Ae. aegypti* populations in mitochondrial NADH dehydrogenase subunit 4 (ND4) gene sequence data (Fig. 1). The four models – panmixia, full migration, stepping-stone migration, and complete isolation – represent different hypotheses of population connectivity and can thus inform the topology of network models of dengue spread (Fig. 2).

The panmixia model represents a hypothesis of high and constant exchange of genes across sites (*Nm* ≫ 1), with all sites behaving as a single, large, random-mating population. The full migration model states that all populations are directly exchanging migrants, but at a level allowing for some genetic drift within sites; under passive, human-mediated dispersal, this means that transportation networks are allowing for gene exchange, either directly (e.g., two sites connected by road) or indirectly (e.g., two sites connected by a third site, such as river trade switching to a road network at a certain node). The stepping-stone model implies that migrants are only exchanged among spatially neighboring populations; here, gene flow occurs only between sites that are adjacent along the transport network (e.g., two harbor cities between which ships may move without stopping). Finally, the complete isolation model represents the hypothesis that gene flow across sites is nonexistent.

The data cover major cities in the Brazilian Amazon and regions in the Americas; these nodes can be connected by land (roads and trains), water (rivers and sea), and air transport edges. *A priori* there is little evidence to suggest complete isolation. On the other hand, it is plausible that gene flow approaches panmixia at the Amazon scale and possibly full migration at the continental scale. This is because of the increase in commercial traffic as a result of globalization of trade (Young et al. 2006; Stoddard et al. 2009). Explicitly testing the support for each model will allow us to take an important step in informing network models for *Ae. aegypti* and dengue.

## Material and Methods

### DNA sequence database

A total of 3103 published ND4 sequences, 322 bp in length, from *Ae. aegypti* sampled in the Americas were obtained from GenBank (Table S1; Fig. 1). To this database, we added 83 new and unpublished sequences (for a total 3186 sequences) obtained from larvae collected in the Amazon cities of Manaus (Amazonas State) by Ríos-Velásquez et al. (2007) and Boa Vista (Roraima State) by Codeço et al. (2009).

We isolated DNA from the 83 samples using the Qiagen DNeasy Tissue Kit following the manufacturer’s protocol (Qiagen, Inc., Valencia, CA, USA). Amplification of the target sequence followed the protocol described by Gorrochotegui-Escalante et al. (2000). Consensus sequences were obtained using GeneDoc 2.6.002 (Nicholas et al. 1997), verified using BLAST (Altschul et al. 1990), and checked for missense mutations or stop codons that would affect the open reading frame using MEGA 4.1 (Tamura et al. 2007). Sequences were aligned manually.

Samples spanned seven countries across the Americas and two major regions within Brazil. To simplify our models, we grouped samples from USA (*N* = 4), Mexico (*N* = 1972), and Central America (*N* = 7), as our focus was on examining broader, continental-scale connections. A separate analysis of these samples suggested they are panmictic; thus, all reported results at this scale refer to the complete sample Mexico/Central America/USA group. Within Brazil, we split samples from the Amazon basin and the southeast, and grouped samples from Rio Branco and Porto Velho within the Amazon basin.

### Data quality control

Nuclear mitochondrial pseudogenes (NUMTs) are nuclear insertions of mitochondrial genes that evolve independently of the mitochondrial genome. Samples containing mixtures of mitochondrial DNA (mtDNA) and NUMT sequences are expected to significantly affect the outcome of genealogy- and frequency-based analyses (Triant and DeWoody 2009). This is because mtDNA and NUMTs have separate genealogies and thus evolutionary history. To minimize the occurrence of NUMTs in our sample, we compiled a list of previously verified mtDNA haplotypes by Hlaing et al. (2009) and Black and Bernhardt (2009)(Data S1). We subsequently removed all sequences within our database that did not match one of these haplotypes. Removing potentially or known non-mtDNA sequences from mtDNA datasets is highly recommended, even though some subjectivity may be involved in the procedure (Yao et al. 2009).

### Descriptive statistics and tests of assumptions

Using the sequences that passed the NUMT filter, we calculated the number of segregating sites (*S*) and nucleotide diversity (*π*), and estimated haplotype diversity (*H*; Table S2) for each population at each sampling scale using DnaSP 5.10.01 (Librado and Rozas 2009).

We used Migrate-N 3.2.6 (Beerli 2006, 2009) for our coalescent-based analysis. The implementation of the coalescent in Migrate-N assumes neutral evolution, no recombination, and that population sizes, time since isolation, and number of demes are finite and have remained constant for at least the previous 2*N*_*ef*_ generations (two times the effective female population size). In addition, Migrate-N assumes an F84 model of nucleotide substitution (Kishino and Hasegawa1989; Felsenstein and Churchill 1996) with or without gamma-distributed variable substitution rates across sites.

To check the neutrality assumption, we tested for departures of the site frequency spectrum from the neutral expectation with Tajima’s (1989)*D* and Fu and Li’s (1993)D* and F* statistics using DnaSP. As these tests are sensitive to demographic effects (Nielsen 2001), we also tested the hypothesis that the ratio of nonsynonymous to synonyms substitutions was different from 1 (*dN*/*dS* ≠ 1) by comparing ND4 sequences from *Ae. aegypti* (GenBank accession number YP001649170.1) to the closely related species *Ae. albopictus* (YP194919.1) as described by Bielawski and Yang (2005).

The hypothesis that *dN*/*dS* ≠ 1 was tested against the null (*dN*/*dS* = 1) using a chi-squared likelihood ratio test (*α* = 0.05, 1 df). Log-likelihood values for each hypothesis were calculated using the codeml package of PAML4 (Yang 2007) by setting *ω* = 1 for the null hypothesis and allowing the program to maximize *ω* for the alternative hypothesis. In either case, we set PAML4 to use the maximum-likelihood estimate (MLE) of the transition/transversion ratio (*κ*) and used an F61 codon substitution model (Yang and Bielawski 2000). The test is based on a maximum-likelihood estimation of the ratio of synonymous to nonsynonymous amino acid changes across the protein sequence (Goldman and Yang 1994). The F61 codon substitution model describes the probabilities of any particular codon changing to another, allowing for all nonstop codon substitutions to have a unique probability of occurrence.

We tested the assumption of constant population size with Ramos-Onsins and Rozas (2002)*R*_*2*_ and Fu’s (1997)*F*_*s*_ using DnaSP. Significance for all statistics was assessed (*α* < 0.05) using empirical distributions based on 10 000 replicates of the neutral coalescent conditional on the observed *S* and sample size, as described by Ramos-Onsins and Rozas (2002).

Regarding separation times between populations, we do not believe them to be ≥2*N*_*ef*_ generations in our study setting. Most *Ae. aegypti* populations in South America were established in the 1970s and 1980s after being all but eliminated earlier in the century (Vasconcelos et al. 1999). If we assume approximately 10–12 generations per year (30 days/generation), about 400–480 generations have passed since 1970. The most in-depth study on *Ae. aegypti* effective population size we are aware of reports wide 95% confidence intervals for most sampling sites in Northern Queensland, Australia (Endersby et al. 2011). For instance, for one town, the estimate is reported to be between 29 and infinity, with a mode of 103. Estimates improved when samples were grouped into larger interconnected urban areas. The largest of which, Townsville (Australia) and surroundings, is smaller than our smallest urban center, Rio Branco (Acre, Brazil). In this grouping, the effective population size estimate was reported as 623 (95% CI = 40 to >10 000). Given the preference of the mosquito for urban sites, it is likely that more urbanized sites will have a higher carrying capacity and thus a larger effective population size than those observed in Northern Queensland.

Thus, because separation times are likely to be <2*N*_*ef*_, at least in the case of Migrate-N, it is not possible to distinguish between gene flow and shared ancestral polymorphism, likely resulting in overestimates of gene flow rates. Nevertheless, simulated data show that the results from this sampler are robust to violations of this assumption when the aim is differentiating among hypotheses about gene flow structure, even though the actual estimate of gene flow may be biased (P. Beerli, personal communication). Finally, we do not believe the data violate the assumption of no recombination; mtDNA is generally accepted as a nonrecombining molecule because of the nature of its inheritance (Birky 1995).

In regard to the nucleotide substitution model used by Migrate-N, we used PAUP* (Swofford 2002) to derive MLEs of its parameters. As mentioned previously, Migrate-N uses an F84 model with or without assuming variable substitution rates. We used a likelihood ratio test to select between constant and variable substitution rates, as described by Goldman and Whelan (2000). For each dataset (as defined by geographic scale of analysis), we first estimated a neighbor-joining (NJ) tree based on a logDet/paralinear distance; we then calculated the MLEs of the transition/transversion rate, nucleotide frequencies, and *α* parameter of the gamma distribution (with four substitution rate categories) based on the NJ tree. Subsequently, we set all parameters of the nucleotide substitution model to the MLE values, and ran a heuristic tree search with an ML criterion, using random stepwise addition of samples and tree bisection–reconnection. We then re-estimated the MLE for each model parameter based on the retained tree(s). We repeated this procedure until the MLEs stabilized between runs and inputted the values of the last run into Migrate-N. In the case of multiple trees, we planned to average MLE values across the trees with the highest log-likelihood values; however, only one tree was found in each case.

### Coalescent-based analyses

We used a coalescent-based approach to examine the support of the data for gene flow structure hypotheses at the continental and Amazon scales. At the continental scale, we examined hypotheses of panmixia, full migration, two stepping-stone scenarios, and complete isolation among five regions or countries: (i) southeastern Brazil; (ii) Brazilian Amazon; (iii) Peru; (iv) Venezuela; and (v) Mexico-North America. As stated above, we grouped Mexican, Central and North American samples in a ‘Mexico-North America’ population (Table S1).

In the first stepping-stone scenario, the Brazilian Amazon is connected by land and river with southeastern Brazil, Venezuela, and Peru; in turn, Venezuela and southeastern Brazil are connected by sea to Mexico-North America. The second stepping-stone scenario differs from the first in that there is no connection between southeastern Brazil and Mexico-North America. *A priori*, the first scenario should be favored over the second, as there is a busy shipping lane between southeastern Brazil with Mexico-North America (SEAS BBXX database, Global Ocean Observing System Center, NOAA: http://www.aoml.noaa.gov/phod/trinanes/BBXX/). We believe that any of the scenarios hypothesizing some degree of gene flow at this scale are plausible given the intensification of trade through globalization (Young et al. 2006; Stoddard et al. 2009). Finally, we examine the hypothesis of isolation, which if accepted would discredit the human-mediated dispersal hypothesis.

Similarly, at the Amazon scale we examined panmixia, full migration, stepping-stone, and isolation hypotheses of gene flow among four areas encompassing major urban centers: north (Boa Vista), center (Manaus), east (Belém), and west (Rio Branco-Porto Velho) (Fig. 1). Again, panmixia, full migration and stepping-stone hypotheses are justified under a man-mediated dispersal hypothesis given trade intensification and the building of transportation networks within Brazil in the last 50–60 years. Regarding the stepping-stone hypothesis, we proposed a star-shaped network in which Manaus is connected to all localities (Fig. 2).

Migrate-N estimates the posterior distribution of mutation-scaled migration (*ℳ*) and mutation-scaled effective population size (Θ) parameters, as well as the marginal likelihood of each gene flow network hypothesis (Beerli 2006). At each spatial scale, gene flow networks were ranked according to their log Bayes factors (LBF) calculated from the Bezier corrected marginal likelihoods of the data given the model. The marginal likelihoods for each model were approximated by thermodynamic integration of the Markov chain Monte Carlo (MCMC) over four spaced heated chains (Metropolis coupled MCMC), as described by Beerli and Palczewski (2010).

Each hypothesis differed in the number of parameters to be estimated, from 1 (panmixia) to 25 (full migration; Fig. 2). These differences affect the length of the MCMC chain required to achieve convergence and thus reliable estimates of the posterior distribution. To accommodate these differences, we ran preliminary runs with the full migration model, which requires the longest chains, and used the same run conditions for all models. In all cases, we ran four parallel static chains with temperatures 1.0, 1.5, 3.0 and 10^6^, with swapping among chains potentially occurring every 10 steps. For each model, we ran 100 replicate runs, with each starting at random points within the domains of the priors. This is a basic feature of Migrate-N that is intended to speed up analyses (P. Beerli, pers. communication). Many smaller chains run in parallel on a computer cluster will produce the same result as one very long chain. Replicate runs are pooled, and the marginal likelihood of each model is calculated from the pooled chains.

We used a uniform prior for *Θ* between 0 and 0.1 and a sampling window of 0.01 on which to generate new proposals; for *ℳ*, we used a uniform prior between 0 and 1000 with a window of 100. The prior on *Θ* is based on the fact that it is a measure of *N*_*ef*_*μ* per base for mtDNA sequences. Hence, a *Θ* of 0.1 means there is 0.1 chance of a new mutation occurring at any given base, or the proportion of bases that are expected to mutate in any given generation. The prior on *ℳ* reflects that we are estimating the proportion of migrants per generation divided by the mutation rate (*m/μ*). Therefore, *ℳ* = 1000 means that *m* is 1000 times larger than *μ*.

Each replicate run had a total of 10^4^ recorded steps at 10^2^ step intervals, for a total of 10^6^ steps, and an initial burn-in of 10^4^ steps. For each replicate run, we analyzed a random subset of 20 and 10 individuals from each population from the continental and Amazon spatial scales, respectively. We assessed convergence by visual inspection of individual chains to ascertain good mixing and lack of trends (Kuhner 2009). We also used the Gelman-Rubin diagnostic to assess convergence across replicate chains, accepting convergence when values were ≤1.20 (Gelman and Rubin 1992). To assess whether we had adequately sampled the posterior distribution, we also required all parameters to have an expected sample size (ESS) ≥10^5^, as calculated by Migrate-N. We plotted chains and calculated the Gelman-Rubin diagnostic using the R (R Development Core Team 2011) package CODA (Martyn et al. 2006).

We report support for each model in terms of LBF units and in terms of the posterior probability. The latter informs on how well any given model is supported relative to other tested models. Furthermore, it is a more easily interpreted measure. The former, however, has historically been used as a criterion in model choice. Kass and Raftery (1995) suggest that a LBF of ≥10 units indicates ‘very strong’ support.

## Results

### Data quality control

In total, we identified 31 haplotypes that were considered to be mtDNA haplotypes by Hlaing et al. (2009) and Black and Bernhardt (2009), with one haplotype exclusive to Asia. Filtering the sequences from our database with these haplotypes removed 292 sequences (12 of which were new sequences generated by us), leaving a total of 2811 sequences in the database (Table S1). The remaining sequences had none of the characteristics typical of NUMTs, while having base frequencies and transition/transversion biases expected for mtDNA sequences (as shown below). Thus, we are confident that the final dataset represents a sample of mtDNA haplotypes from *Ae. aegypti*.

### Descriptive statistics and tests of assumptions

At the continental scale, we observed a total of 30 haplotypes (*h*), with within-population values ranging between 2 and 22 (Table S2). Haplotype diversity was generally high (*Hd* > 0.60) for all populations except Peru (*Hd* = 0.42; Table S2), indicating relatively even haplotype frequencies within populations. Because we only observed two haplotypes in Peru, an *Hd* approximately 0.50 also indicates an even distribution of haplotypes.

At the Amazon spatial scale, we observed a total of 12 haplotypes, with *h* ranging from four to seven within populations (Table S2). Haplotype diversity was not as uniform as seen at the continental spatial scale; Manaus and Rio Branco-Porto Velho had values <0.50, suggesting that some haplotypes were more abundant than others in our sample, while Belém and Boa Vista had values >0.50 (Table S2). Nevertheless, sample sizes do not allow us to confidently estimate number of haplotypes and haplotype frequencies within these localities. Further results for both scales can be found in Data S2.

At the continental spatial scale, Tajima’s *D* and Fu and Li’s D* and F* did not suggest negative selection (Table 1). In four of five populations, however, Tajima’s *D* was significantly larger than expected by the neutral coalescent. The same was observed for Fu and Li’s D* in Peru, and for Fu and Li’s F* in Peru, Venezuela, and Mexico-North America. Assuming demographic stability, theory would indicate that this is evidence for balancing selection. Nevertheless, in all four populations with significantly positive neutrality test indices, significantly positive values were also observed for Ramos-Onsis and Rozas’*R*_*2*_ and/or Fu’s *F* statistics, indicating a potential bottleneck. Thus, the assumption of demographic stability seems to be violated in these populations, as the significantly positive values of neutrality statistics could also be explained by population decline.

**Table 1:** Test of demographic stability and selective neutrality across 322 bp of the *Aedes aegypti* ND4 gene sampled across the Americas

		Demographic stability		Neutrality tests		
			
Scale	Population	*R*_*2*_	*Fs*	*D*	D*	F*
Continent	Mexico-North America	0.15*	7.17*	3.45*	1.00	2.51*
	Venezuela	0.14*	9.82*	2.25*	1.40	2.10*
	Peru	0.21*	14.26*	2.63*	1.44*	2.16*
	Brazilian Amazon	0.18*	1.93	2.16*	0.57	1.35
	Southeastern Brazil	0.15	1.41	1.38	1.01	1.36
Amazon	Boa Vista	0.13	0.31	−0.80	−0.44	−0.60
	Manaus	0.09	0.19	−0.78	0.88	0.44
	Belém	0.22*	0.97	1.75*	1.14	1.50
	Rio Branco-Porto Velho	0.17	0.86	−2.04†	−3.20†	−3.33†

* Values significantly larger than expected by the neutral coalescent (*P* < 0.025).

† Values significantly smaller than expected by the neutral coalescent (*P* < 0.025).

*R*_*2*_ (Ramos-Onsins and Rozas 2002); *Fs* (Fu 1997); *D* (Tajima 1989); and D* and F* (Fu and Li 1993).

At the Amazon spatial scale, observed values for neutrality and demographic stability statistics in Manaus and Boa Vista were within expectations of the neutral coalescent, but those observed in Belém and Rio Branco-Porto Velho were not. Tajima’s *D* for Belém was significantly larger than expected by the neutral coalescent (*P* < 0.025). Yet, once again, the *R*_*2*_ value was also significantly larger (*P* < 0.025), indicating that population decline is also a plausible explanation for the large Tajima’s *D*. In Rio Branco-Porto Velho, we observed values significantly smaller than expected by the neutral coalescent (*P* < 0.025), and no indication of demographic expansion as observed by the values of *R*_*2*_ and *F*s. Thus, it is plausible that negative selection is strong in this region.

Further evidence for negative selection is obtained from the more general results of the pairwise comparison of *Ae. aegypti* and *Ae. albopictus* ND4 genes using PAML. The neutral model (*dN* = *dS*) resulted in a log-likelihood value of −2126.52, while the ML model (in which *ω* is estimated) resulted in a log-likelihood value of −1959.66; a likelihood ratio test suggests that the MLE of *ω* = 0.011 is significantly different (*P* < 0.05) from *ω* = 1.00. The between-species is thus strongly suggestive that ND4 is likely under rangewide negative selection in *Ae. aegypti.*

The MLEs for the F84 nucleotide substitution model parameters suggested unequal base frequencies in line with observations from mtDNA of insects and *Ae. aegypti* (Dueñas et al. 2006; Table S3). Across sampling scales, differences in base frequency MLEs were on the order of 10^−3^ and were considered too small to be reported. Transition/transversion bias was higher at the Amazon scale (8.08) than at the continental scale (3.75). These values are smaller than previously reported for mtDNA (9.0–10.6; Wakeley 1996) and could be a function of different levels of genetic drift or selective pressures occurring at these scales (Wakeley 1996). Our analysis also showed that including among-site rate variation modeled by a gamma distribution provided a better fit of the nucleotide substitution model to the data at both spatial scales (*P* ≪ 0.01 and *P* < 0.05 for continent and Amazon scales, respectively). The shape parameter values are relatively small (*α* = 0.031 and 0.004 for the Amazon and continental scales, respectively), suggesting a high proportion of almost invariant sites and few highly variable sites. This is expected for a gene under strong negative selection and is mirrored in the finding of synonymous mutations at 17 of 22 segregating sites.

### Coalescent-based analyses

Visual inspection of the MCMC chains showed no trends and good mixing, suggesting convergence (data available upon request). Values of the Gelman-Rubin diagnostic were all ≤1.11, with the majority of values ≤1.05. Expected sample size values were all ≥3.9 × 10^5^, with a maximum of 2.19 × 10^6^. While it is always difficult to conclude with certainty that convergence of the MCMC has occurred, it is possible to infer with some degree of certainty that convergence has not occurred (Kuhner 2009). By having suitably high ESS values, along with multiple (100) replicates with evidence for convergence across chains and visual inspection of individual chains suggesting good mixing and a lack of trends, we are confident that we generated a good sample of the posterior distribution of the parameters of interest.

At the continental scale, stepping-stone model 1, which includes a connection between Mexico-North America and southeastern Brazil (Table 2), had the highest support from the data. This model had a posterior probability of 0.99 and had a LBF of −33.58 relative to the second-ranking model, stepping-stone 2, which did not feature the Mexico-North America/southeastern Brazil connection. At the Amazon spatial scale, the full migration model had the highest support from the data (Table 2), with a posterior probability of 0.94. However, the LBF when compared to the panmixia model was −5.76. In the Kass and Raftery (1995) scale, this provides some positive support for the full-migration model, but should not be considered definitive, as discussed for instance in Clarke and Middleton (2008). Posterior densities for each parameter of the best ranking models are reported in Fig. S1 and S2.

**Table 2:** Marginal log-likelihood values, log Bayes factor and ranks of continental and Amazon-scale gene flow network models

Scale	Model (*N* parameters)	Marginal log-likelihood	Log Bayes factor	Posterior probability	Rank
Continent	Stepping-stone 1 (15)	−692.40	0.00	0.99	1
	Stepping-stone 2 (13)	−709.19	−33.58	10^−8^	2
	Full migration (25)	−714.40	−44.00	10^−10^	3
	Panmixia (1)	−725.38	−65.96	10^−15^	4
	Isolation (5)	−947.11	−509.42	0.00	5
Amazon	Full migration (16)	−548.71	0.00	0.94	1
	Panmixia (1)	−551.59	−5.76	0.05	2
	Stepping-stone (10)	−574.02	−50.62	10^−12^	3
	Isolation (4)	−657.24	−217.06	10^−48^	4

## Discussion

Our objective was to test the data support for alternative gene flow network models in the dengue vector, *Ae. aegypti*, at two different spatial scales and thus inform epidemiological network models of *Ae. aegypti* and dengue spread. At the continental spatial scale, the model with the highest support from the data (0.99 posterior probability) suggests a significant link between southeastern Brazil and Mexico-North America, along with connections between Mexico-North America and Venezuela; Venezuela and the Brazilian Amazon; and the Brazilian Amazon, Peru, and southeastern Brazil. The LBF to the second ranking model (−33.58) gives us confidence that, among the tested models, this is the one that most likely represents the process that generated the data (Table 2).

At the Amazon scale, we examined connectivity among four major urban centers: Rio Branco-Porto Velho in the west; Manaus in the center; Belém in the east; and Boa Vista in the north. At this scale, a full-migration model of connectivity had the highest support from the data (0.94 posterior probability), suggesting that all sampling sites are exchanging migrants. However, the marginal log-likelihood difference between this model and the panmictic model (LBF = −5.76) suggests that more data are required to confidently distinguish between them (Table 2). Alternatively, this could reflect seasonal variation in gene flow networks, with higher connectivity during the wet season when compared to dry seasons. If so, a mixed model would be more appropriate. Nevertheless, both models imply a significant degree of *Ae. aegypti* gene flow across Amazonian urban centers. The isolation models at either spatial scale, on the other hand, produced the worst fit to the data; while this result was not unexpected, it underscores that locally isolated *Ae. aegypti* control efforts are unlikely to succeed, implying that concerted region-wide programs are needed to contain the spread of *Ae. aegypti* and, consequently, the spread of dengue.

Our dataset violates a number of assumptions of the coalescent model employed in Migrate-N. However, we do not feel that this invalidates our results, because we did not wish to obtain parameter estimates for *Θ* or *ℳ* but rather wished to examine the support of the data in alternative gene flow network models (Beerli and Palczewski 2010). Below we argue our point for each assumption.

Violation of selective neutrality because of strong negative selection should not change the relative rank of gene flow networks. As the selective pressure at this locus is likely to be uniform across the species range, affecting all populations equally, it should not skew gene flow estimates as would be expected if selective pressures differed among populations (Charlesworth et al. 1997; McCracken et al. 2009). Furthermore, negative selection will affect the length of gene genealogies (i.e., coalescence happens <2*N*_*ef*_ generations) but not the topology (Hudson and Kaplan 1995). Therefore, while the actual estimate of gene flow may be an underestimate, the relative difference between gene flow networks will not be affected.

Unfortunately, the effects of violations of demographic stability and time since divergence on the performance of Migrate-N to rank gene flow network models are not as clear. Simulations by Beerli (2009) show that the effect of violating demographic stability on parameter estimates depends on the magnitude and the timing of demographic events. Our results suggest recent bottlenecks in Peru, Venezuela, Mexico-North America, and Belém, which is consistent with a history of dengue prevention programs relying exclusively on vector control, which has likely caused several cycles of local extinction followed by re-colonization (Figueiredo 2003). Thus, parameter estimates may be biased in our analyses; yet, the effect on the ranking of gene flow networks is still unknown. However, as long as there is some gene flow across populations, as explained below in regard to population divergence, Migrate-N seems robust to such violations.

If population divergence occurred <2*N*_*ef*_ generations ago, populations are likely to share genetic variation because of both migration events and shared ancestry. Because Migrate-N assumes that shared variation is attributed to migration, this can result in inflated estimates of migration. However, as long as there is some migration, Migrate-N is robust to violations of this assumption and should be able to correctly infer gene flow structure in most cases at divergence times of at least *N*_*ef*_/2 generations or even more recent times if migration rate is high (see http://popgen.sc.fsu.edu/Migrate/Blog/Entries/2010/8/15_Violation_of_assumptions%2C_or_are_your_migration_estimates_wrong_when_the_populations_split_in_the_recent_past.html). Thus, it seems that the crucial parameter determining the ability of Migrate-N to infer the correct gene flow network given presence of ancestral variation is the ratio of shared variation because of migration to shared variation because of ancestry. In the case of *Ae. aegypti*, high levels of gene flow across major urban centers are plausible and generally accepted (Urdaneta-Marquez and Failloux 2011); while gene flow measures based on *F*_ST_ are not generally to be trusted, there are at least two independent sources of evidence that substantiate this belief: (i) the rapid spread of *Ae. aegypti* across the globe starting in the 15th century, and the rapid re-infestation of New World regions once eradication programs were discontinued in the 1950s (Groot 1980; Gubler 1989); and (ii) the continued reports of mosquitoes and their larvae on boats, airplanes, trains and trucks (Lounibos 2002).

Therefore, in light of the current understanding of Migrate-N, the coalescent, and the evidence on *Ae. aegypti* spread, we are confident that our results are no more impacted by our data violating the coalescent model implemented in Migrate-N than if we had analyzed them using an allele-frequency framework. Yet, because we take a Bayesian coalescent approach, we are able to explicitly test different hypotheses of gene flow structure by examining their fit to the data. Future work in Migrate-N would greatly benefit from adding coalescent models that explicitly account for selection, demographic instability and nonequilibrium divergence times. Such models would allow us to properly gauge the effects of the violations of the above assumptions in parameter estimates and model selection. In the absence of a model that incorporates selection, a simulation study should be carried out to fully test the robustness of Migrate-N to the violation of selection. Software such as SFS_CODE (Hernandez 2008) could be used to generate the necessary samples. Alternatively, the complex history of *Ae. aegypti* might be ideally suited for analyses using novel Approximate Bayesian Computation methods (Beaumont 2010).

If we accept that Migrate-N is correctly ranking gene flow models, how do the results inform on the man-mediated dispersal hypothesis? As mentioned previously, our gene flow network hypotheses were derived from presumed connections between sampling sites along different transportation networks. At the continental scale, at least in some cases, there is good evidence in support of these hypotheses; for instance, land connection between Venezuela and Boa Vista (northern Brazilian Amazon) is suggested by the presence in Boa Vista of dengue serotypes previously only recorded in Venezuela (Lourenco-De-Oliveira et al. 2004; Figueiredo et al. 2008; Codeço et al. 2009), and river connection between Peru and the Brazilian Amazon is supported by pupae surveys on boats (Morrison et al. 2006).

On the other hand, the connections of Venezuela and southeastern Brazil with Mexico-North America, as well as the connection between the Brazilian Amazon and southeastern Brazil, require more data. The presence of major shipping lanes connecting the Gulf of Mexico with Venezuela and Brazil, the fact that shipping is largely responsible for the spread of *Ae. aegypti* globally, and genetic data suggesting higher genetic variability in port towns in Asia (Hlaing et al. 2010), southeastern Brazil (Paduan and Ribolla 2008), and Belém (this study), however, suggest that this is a plausible scenario.

The connection between the Brazilian Amazon and southeastern Brazil is often implied (Vasconcelos et al. 1999), in particular by the history of *Ae. aegypti* re-invasion after the 1955 eradication, which presumably began in southeastern Brazil, reached the central-west and northeastern regions by the mid-1980s, and finally all Brazilian states by 1998 (Figueiredo 2003). The second half of the 20th century also saw the building of major highways linking the south and southeastern regions of the country with the northeast and northwestern regions (e.g., the BR364 from São Paulo to Acre, and the BR153 from Rio Grande do Sul all the way to Belém; Fig. 1). Thus, at the continental scale, there is some evidence to support a correlation between the inferred gene flow links and transportation networks.

At the Amazon scale, Porto Velho, Manaus and Belém are all connected by river (Fig. 1), and Amazonian riverboats are a known a mode of dispersal of *Ae. aegypti* larvae (Morrison et al. 2006). Boa Vista and Manaus are connected by a major highway (BR174) that has been hypothesized as a route for *Ae. aegypti* and dengue spread into Brazil from Venezuela, the Caribbean, Suriname and the Guyanas (Lourenco-De-Oliveira et al. 2004; Figueiredo et al. 2008; Codeço et al. 2009). Finally, we treated Rio Branco and Porto Velho as one site because of the proximity between the two cities (approximately 450 km along the BR364 highway), and because all land commercial transport between Rio Branco and the rest of Brazil must, given the current highway system, go through Porto Velho (Fig. 1).

Direct connections between sites without land or river links (e.g., Boa Vista and Rio Branco-Porto Velho or Belém), as suggested by the full-migration model, may reflect an intensification of regional air traffic; however, indirect connections driven by the transportation of goods across multiple nodes in the network are also plausible. Therefore, the current transport network in the Brazilian Amazon does not contradict the gene flow structure inferred by our analysis and provides support for the hypothesis that such networks are contributing to *Ae. aegypti* passive dispersal in the region.

Our results, it should be noted, only reflect female dispersal because mtDNA is maternally inherited. A comparison of nuclear single-nucleotide polymorphisms (SNPs) and ND4 genes across Venezuela suggests spatial structure in ND4, but no structure in SNPs (Urdaneta-Marquez et al. 2008). Such a pattern is consistent with sex-biased dispersal, with the female being the philopatric sex (Melnick and Hoelzer 1992). If this is indeed the case, our results reflect the minimum gene flow network.

Across scales, different gene flow networks models were favored by the data. Our observation of full migration at the Amazon scale suggests that as commercial traffic intensifies, the continental stepping-stone gene flow structure may shift to full migration or panmixia. The high levels of connectivity observed at the Amazon scale are expected to facilitate the spread of advantageous mutations (e.g., resistance to insecticides) and potentially increase the costs of control. It is difficult to infer how gene flow networks will be at smaller spatial scales, and thus what to expect in terms of spread and control at these scales. But if the trend is toward increased gene flow at finer scales, then it is expected that at such scales as between cities and some nearby rural areas we might observe full-migration or panmixia. Future work may benefit from more intense and finer spatial scale sampling to evaluate such scenarios.

Even though our data demonstrate gene flow across sampling sites at both spatial scales, and the inferred gene flow networks can be largely explained in terms of the transportation networks, we still do not have direct evidence for the human-mediated dispersal hypothesis. Targeted sampling within urban sites and on trucks, boats, planes, and possibly trains arriving at each city would be necessary to provide a definitive test of the hypothesis. By noting the origin of the vehicles and using genetic data from those sites to match samples, we would be able to make definite links to the role of commercial transport, estimating the contribution of each form of transport to the total influx of *Ae. aegypti* at each locality, as well as the proportional contribution from different source sites.

The models we tested do not represent an exhaustive search of all possible models. For instance, it is plausible that gene flow is not symmetric across sites and, in some cases, it may be largely unidirectional. This may occur if vector control measures differ between sites. We have avoided more complex models because a one-locus dataset has little power to correctly rank asymmetrical models (Beerli and Palczewski 2010). In such cases, information on multiple nuclear loci is needed to confidently rank gene flow networks and estimate gene flow levels. Thus, future work should focus on recently available nuclear loci (e.g., Lovin et al. 2009), which will allow us to take full advantage of the coalescent model (Nichols 2001; Brito and Edwards 2009). Multiple loci will help minimize coalescent variance and increase confidence in estimates of directionality and amount of gene flow between nodes. However, this does not decrease the value of our analyses. In simpler network models, the connection pattern among nodes is one of the most essential parameters (Proulx et al. 2005). Hence, we consider the results presented here an important first step in the direction of more detailed epidemiological network models.

In conclusion, our results shed light on the node-edge structure of *Ae. aegypti* gene flow networks at two spatial scales that are relevant to public health planning and management; they can also contribute to the development of network-based models of *Ae. aegypti* population dynamics and dengue spread at these spatial scales.

## Data archiving statement

Raw data for this file are available on GenBank and as supplementary online material (Data S1). See Table S1 for GenBank Accession numbers.

## Supporting information

**Figure S1.** Posterior density for all 15 parameters of the stepping-stone 1 model at the continental scale.

**Figure S2.** Posterior density for all 16 parameters of the full-migration model at the Amazon scale.

**Table S1.** Sample database.

**Table S2.** Summary statistics for 2183 322 bp ND4 mitochondrial sequences across two spatial scales in the Americas.

**Table S3.** Maximum likelihood estimates of the parameters of the F84 + Γ for each of the examined datasets obtained using PAUP*.

**Data S1.** Sequences identified as NUMTs (FASTA format).

**Data S2.** Additional results, including Tables S1, S2 and S3.
