# Supplementary figures and images for: Gene flow networks among American Aedes aegypti populations
Source: Evol Appl. 2012 Nov;5(7):664–76. doi: 10.1111/j.1752-4571.2012.00244.x (PMC3492893; doi:10.1111/j.1752-4571.2012.00244.x)

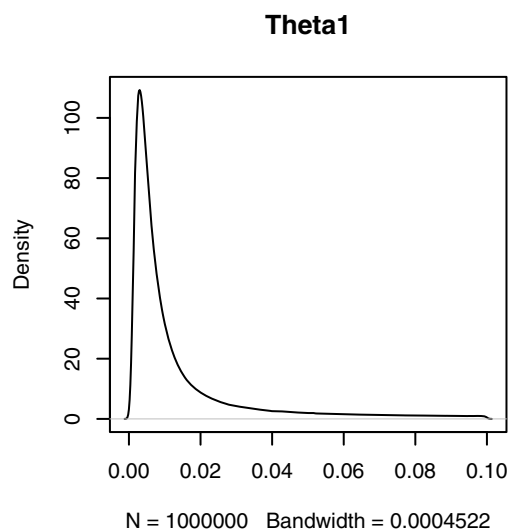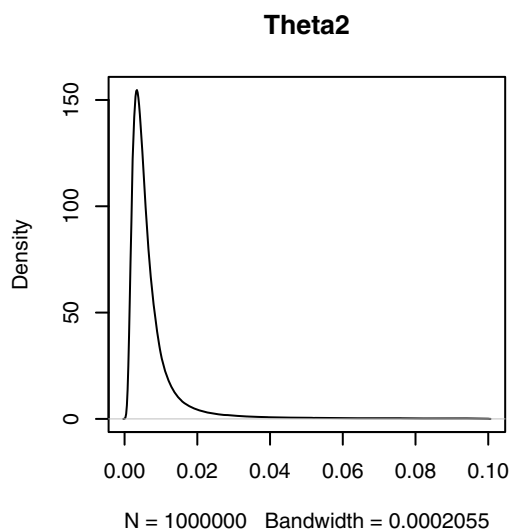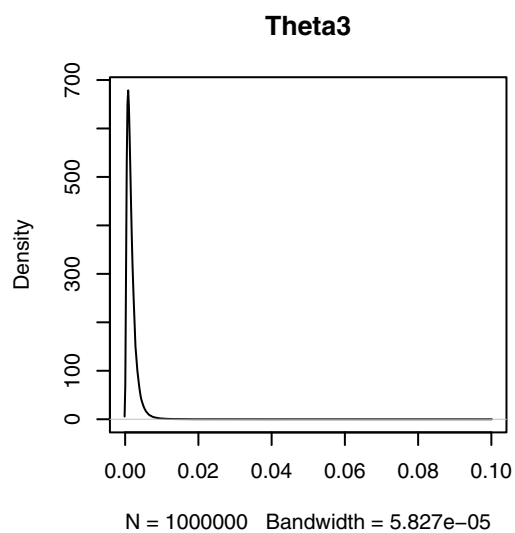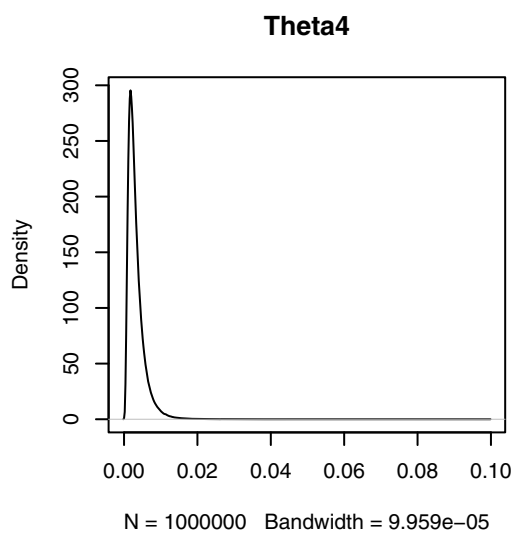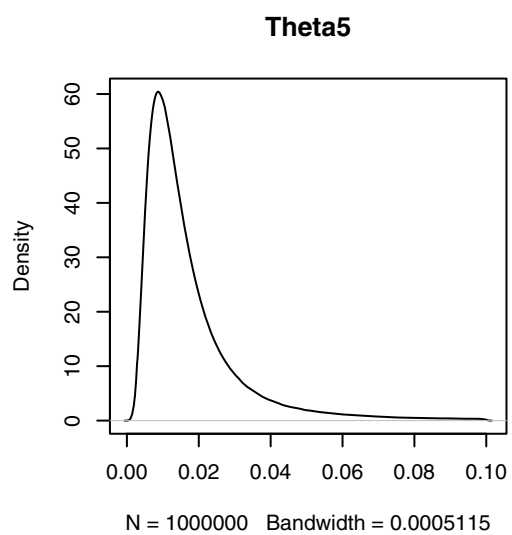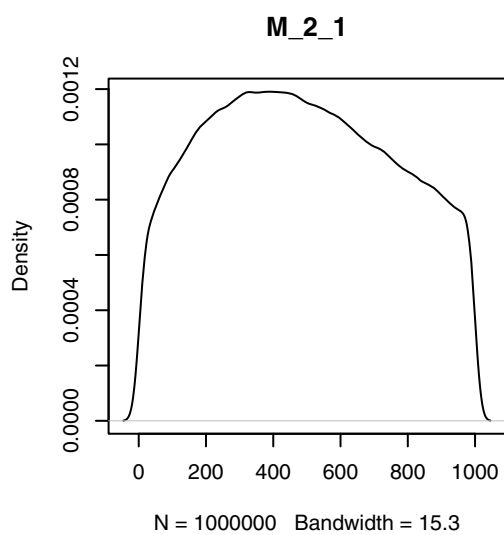

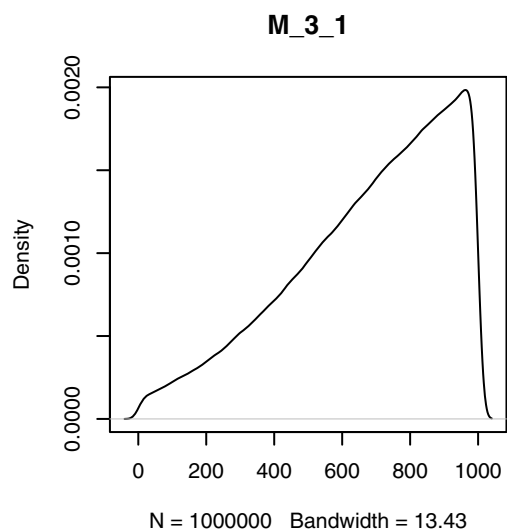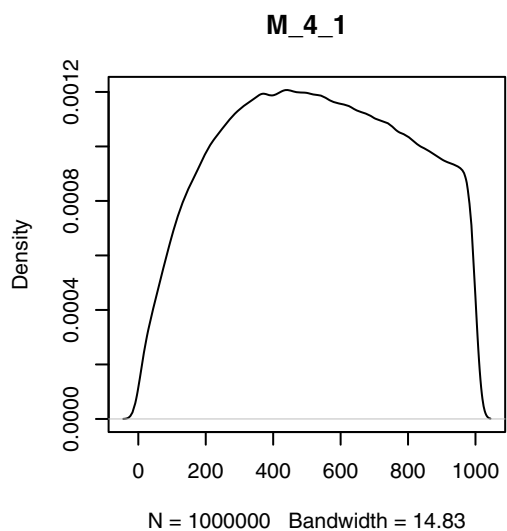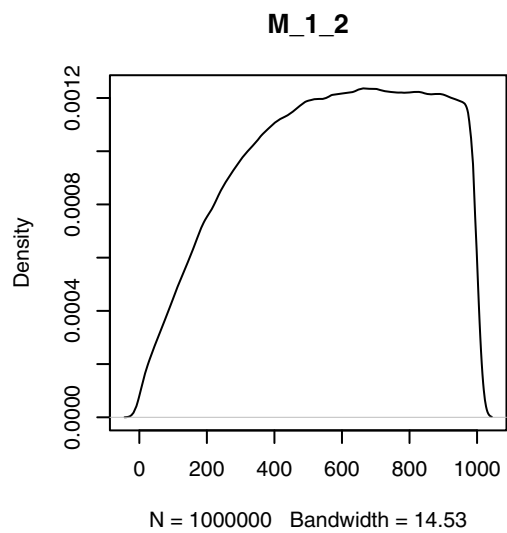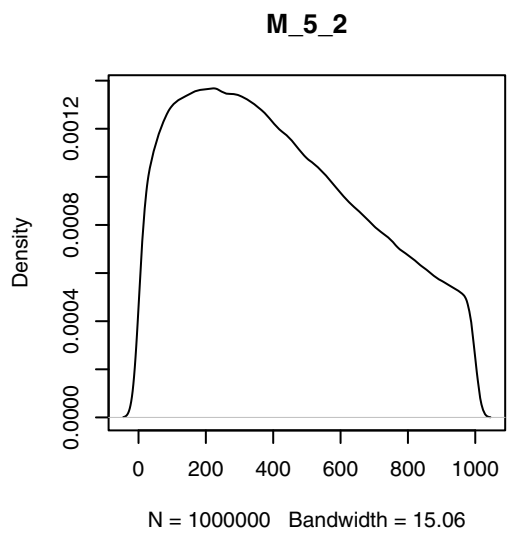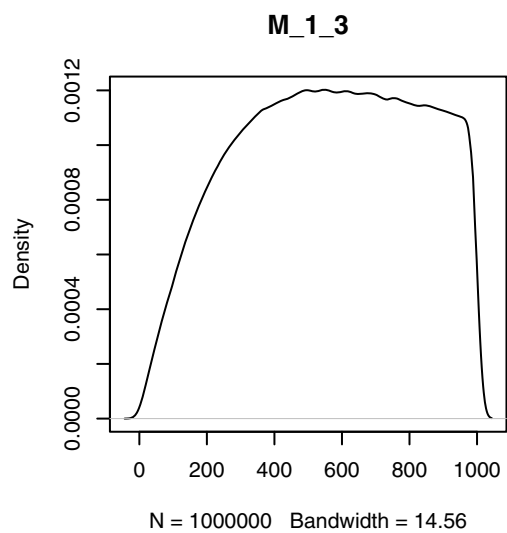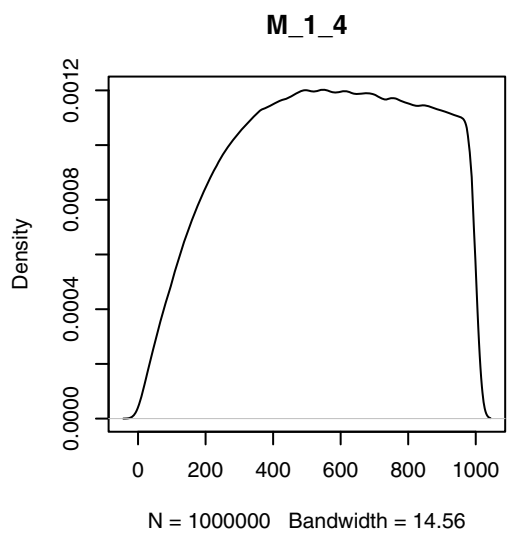

**M\_5\_4**

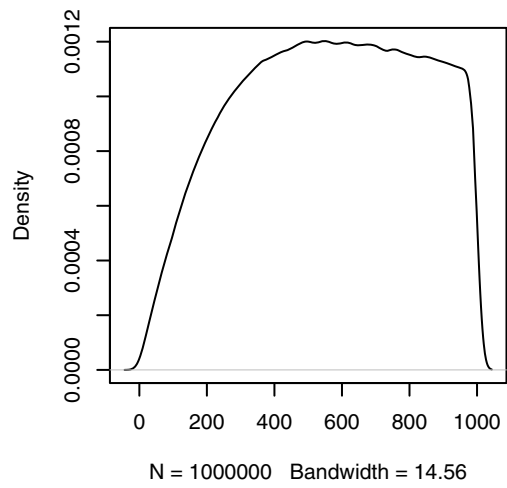

**M\_2\_5**

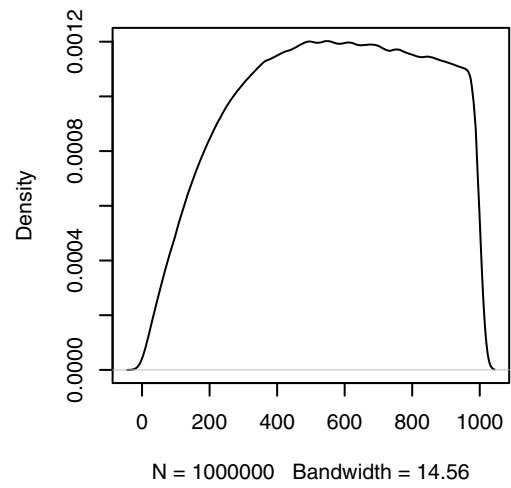

**M\_4\_5**

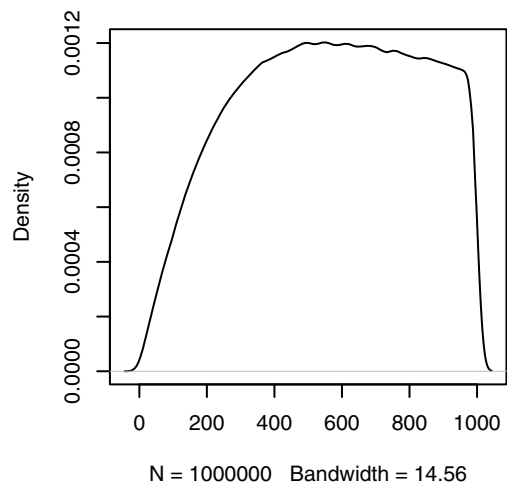

Supplement: Supplementary file 1 [file eva0005-0664-SD1.pdf]

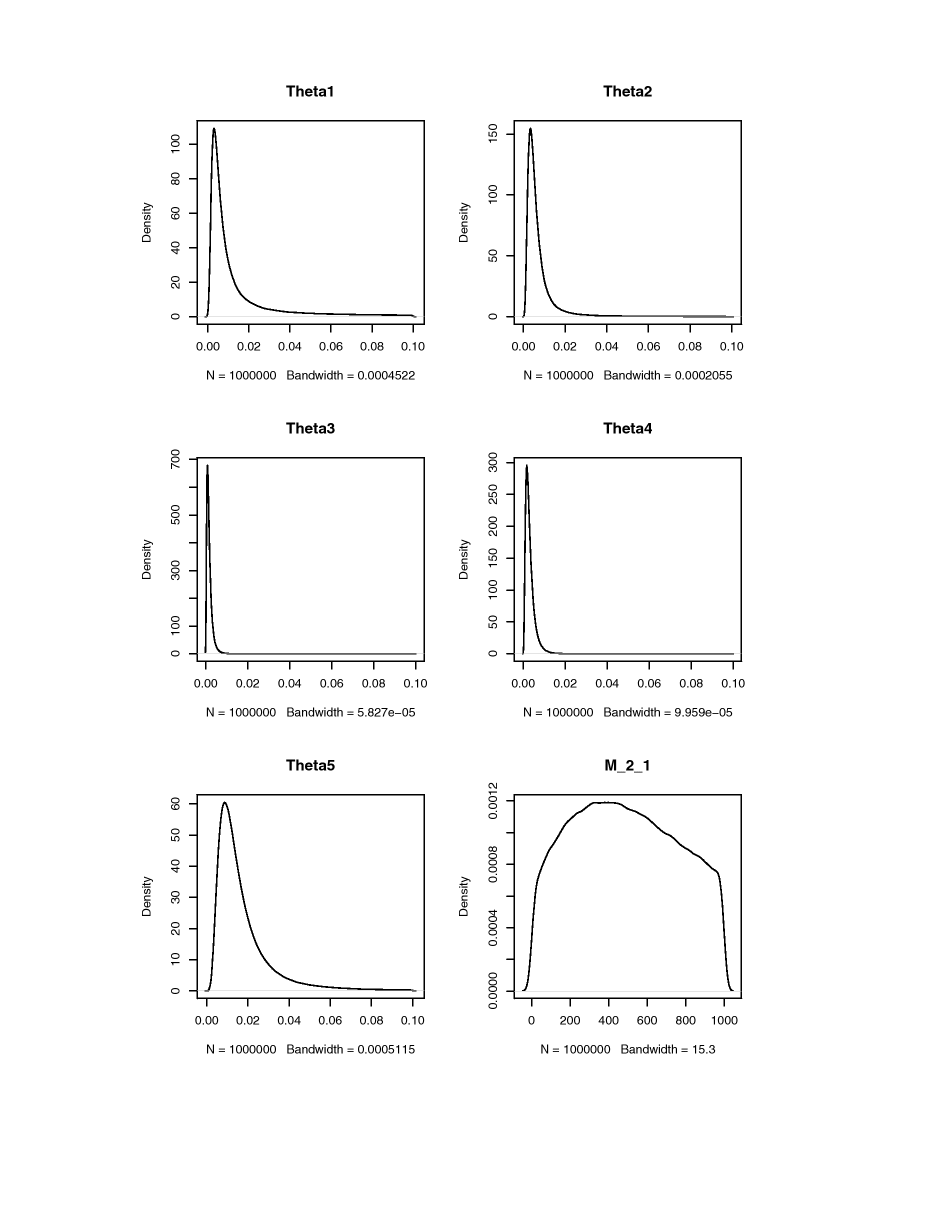

Supplement: Supplementary file 3 [file eva0005-0664-SD7.png]

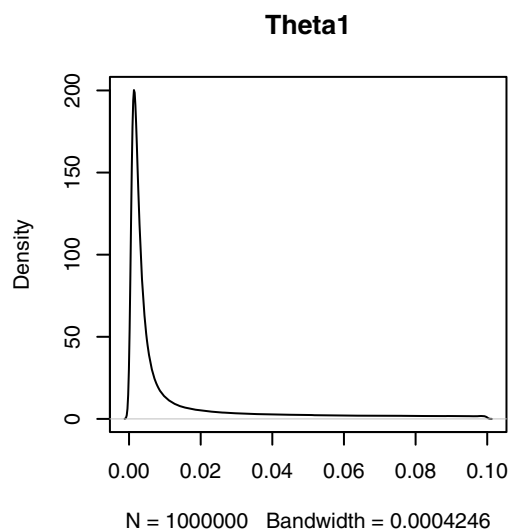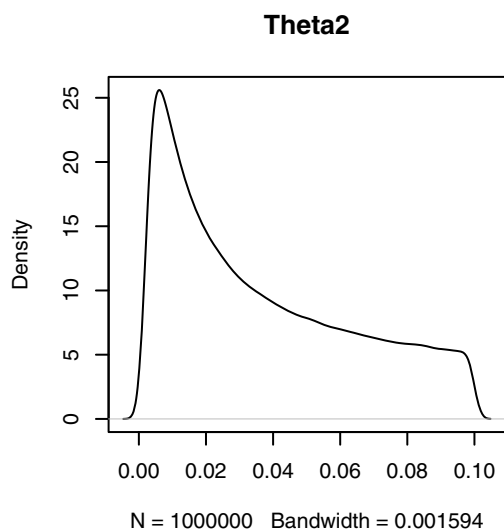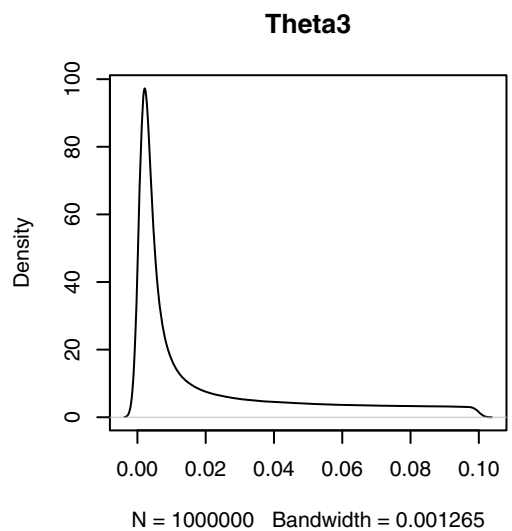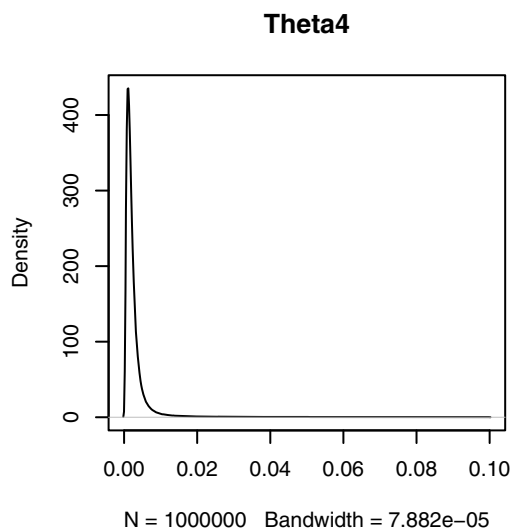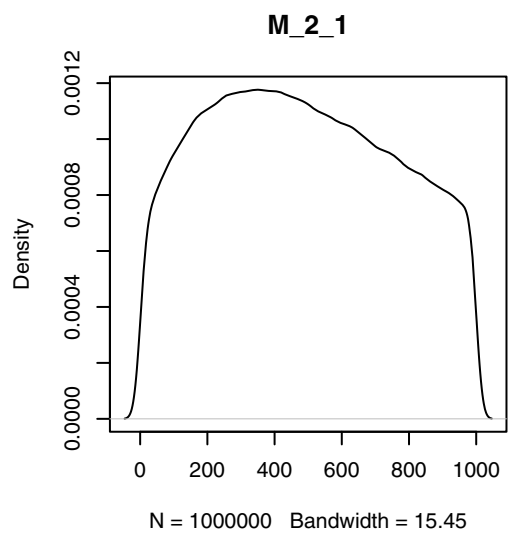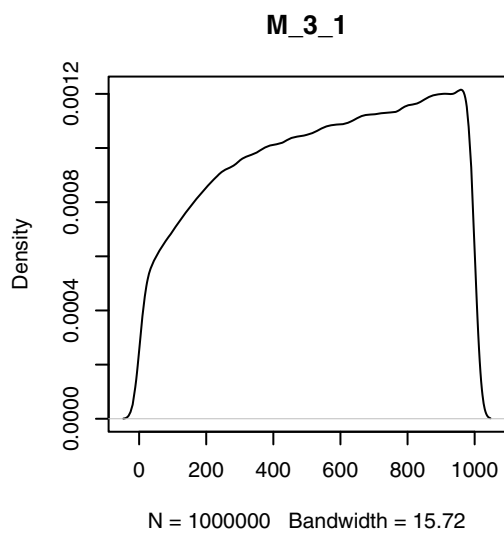

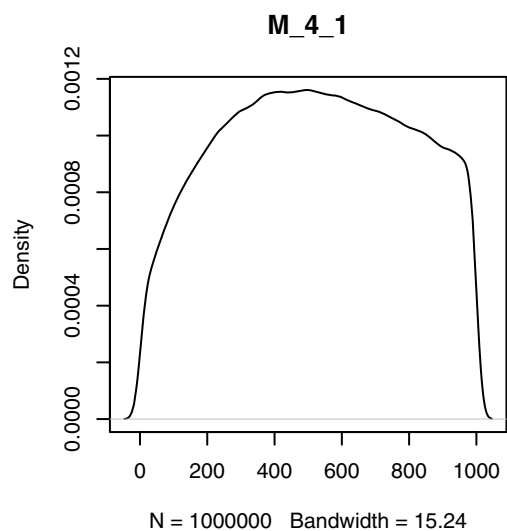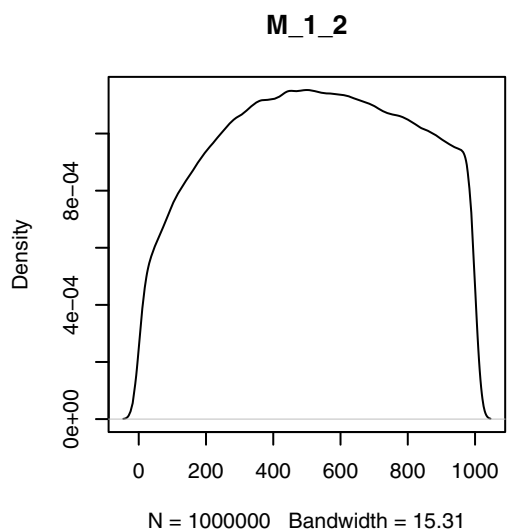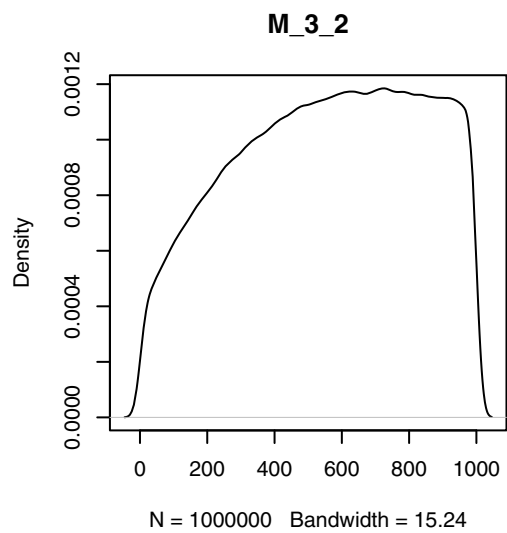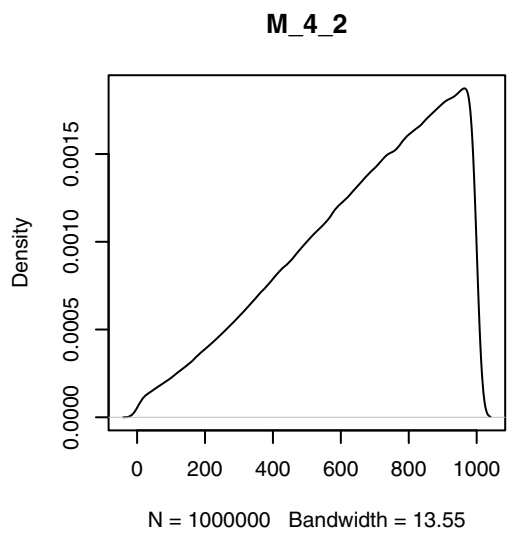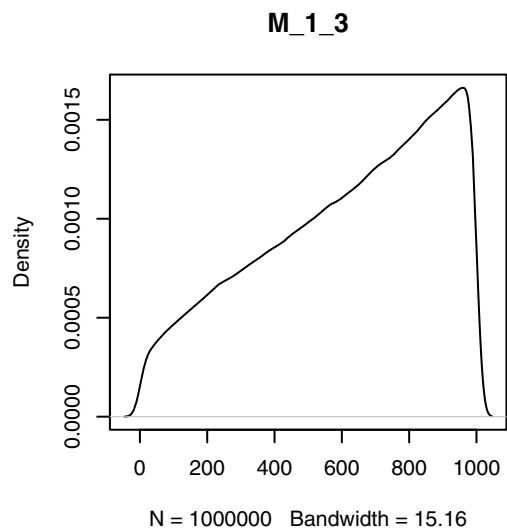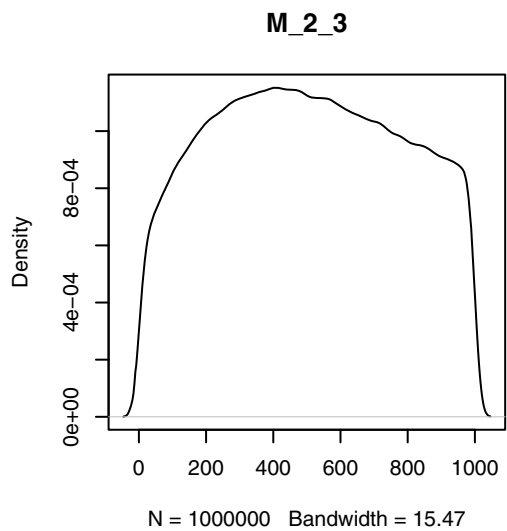

**M\_4\_3**

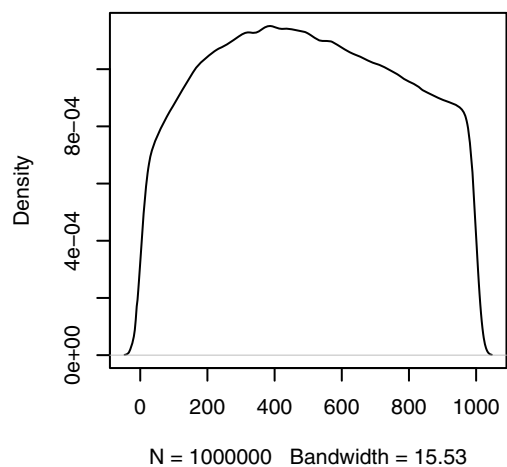

**M\_1\_4**

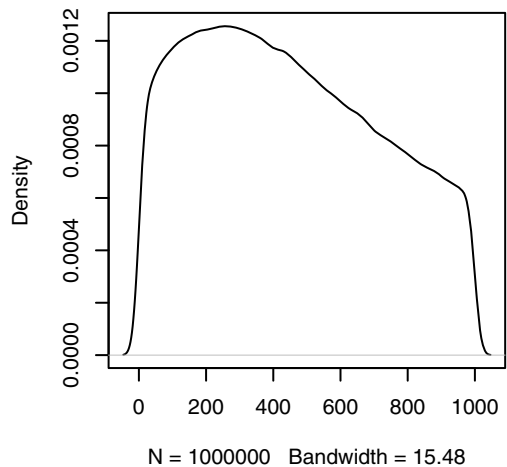

**M\_2\_4**

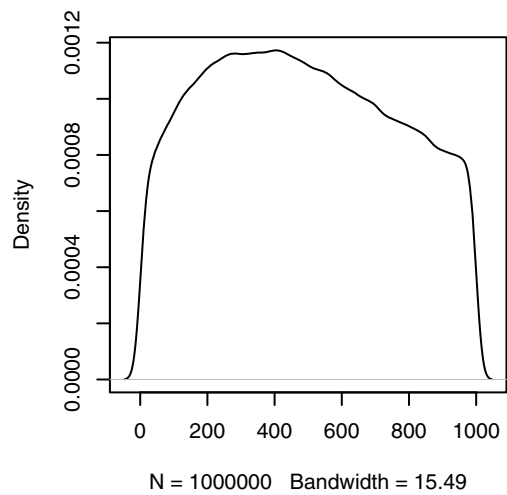

**M\_3\_4**

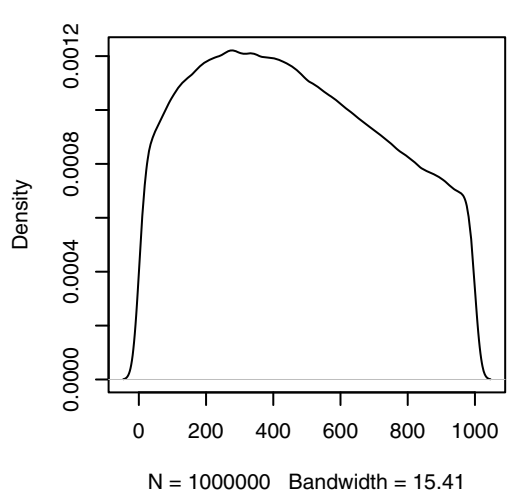

Supplement: Supplementary file 4 [file eva0005-0664-SD3.pdf]

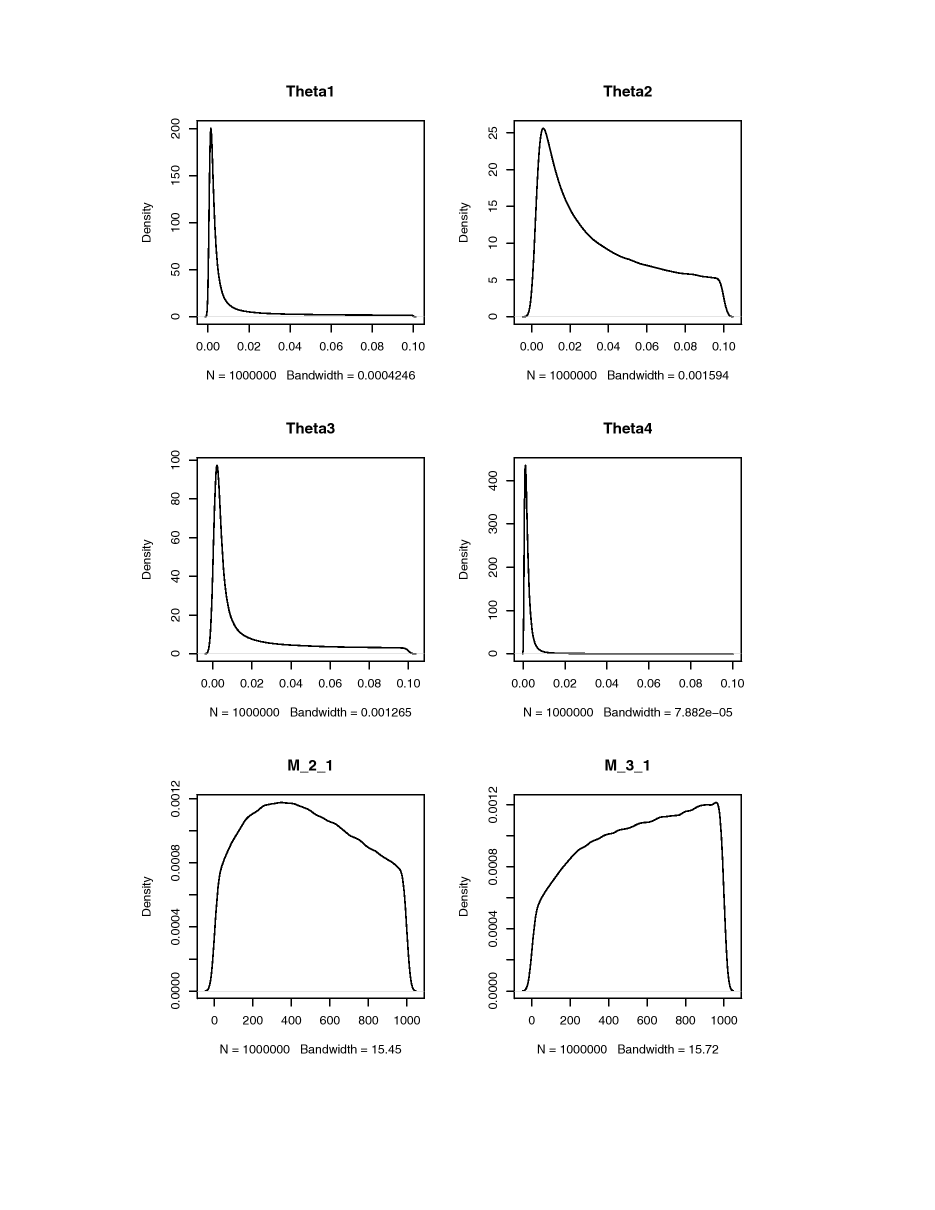

Supplement: Supplementary file 6 [file eva0005-0664-SD8.png]
