# Supplementary material for: Gene flow networks among American Aedes aegypti populations
Source: Evol Appl. 2012 Nov;5(7):664–76. doi: 10.1111/j.1752-4571.2012.00244.x (PMC3492893; doi:10.1111/j.1752-4571.2012.00244.x)
Supplement: Supplementary file 8 [file eva0005-0664-SD6.doc]

Supplementary information

**Database**

Table S1. Sample database

|  | **Continent** | | | | | **Amazon** | | | |  |  |
| --- | --- | --- | --- | --- | --- | --- | --- | --- | --- | --- | --- |
| **Hap** | **BrAM** | **SEBr** | **PE** | **VZ** | **M-NA** | **MAO** | **BEL** | **BV** | **RB-PV** | **GenBank** | **Citation** |
| Hap_1 | 0 | 0 | 0 | 0 | 283 | 0 | 0 | 0 | 0 | AF334858.1 | Gorrochotegui-Escalante et al. (2002) |
| Hap_2 | 0 | 0 | 0 | 0 | 214 | 0 | 0 | 0 | 0 | AF334851.1 | Gorrochotegui-Escalante et al. (2002) |
| Hap_3 | 0 | 0 | 0 | 0 | 93 | 0 | 0 | 0 | 0 | AF334852.1 | Gorrochotegui-Escalante et al. (2002) |
| Hap_4 | 0 | 0 | 0 | 0 | 139 | 0 | 0 | 0 | 0 | AF334854.1 | Gorrochotegui-Escalante et al. (2002) |
| Hap_5 | 19 | 11 | 36 | 94 | 330 | 1 | 2 | 0 | 16 | EU650409.1 | Lima Jr and Scarpassa (2009) |
| Hap_6 | 9 | 47 | 15 | 0 | 355 | 0 | 6 | 2 | 1 | EU650414.1 | Lima Jr and Scarpassa (2009) |
| Hap_7 | 0 | 0 | 0 | 0 | 130 | 0 | 0 | 0 | 0 | AF334864.1 | Gorrochotegui-Escalante et al. (2002) |
| Hap_8 | 0 | 0 | 0 | 0 | 55 | 0 | 0 | 0 | 0 | AF334850.1 | Gorrochotegui-Escalante et al. (2002) |
| Hap_9 | 0 | 0 | 0 | 0 | 154 | 0 | 0 | 0 | 0 | AF334859.1 | Gorrochotegui-Escalante et al. (2002) |
| Hap_10 | 30 | 2 | 0 | 99 | 9 | 22 | 1 | 7 | 0 | EU650415.1 | Lima Jr and Scarpassa (2009) |
| Hap_11 | 3 | 3 | 0 | 6 | 8 | 0 | 2 | 0 | 1 | AY906852.1 | Paduan and Ribolla (2008) |
| Hap_12 | 6 | 0 | 0 | 10 | 11 | 1 | 1 | 0 | 4 | EU650407.1 | Lima Jr and Scarpassa (2009) |
| Hap_13 | 1 | 3 | 0 | 0 | 0 | 0 | 1 | 0 | 0 | EU650412.1 | Lima Jr and Scarpassa (2009) |
| Hap_14 | 1 | 0 | 0 | 85 | 0 | 0 | 1 | 0 | 0 | EU650413.1 | Lima Jr and Scarpassa (2009) |
| Hap_15 | 1 | 1 | 0 | 331 | 1 | 0 | 0 | 1 | 0 | DQ176835.2 | Bracco et al. (2007) |
| Hap_16 | 1 | 3 | 0 | 0 | 55 | 1 | 0 | 0 | 0 | DQ176840.2 | Bracco et al. (2007) |
| Hap_17 | 1 | 0 | 0 | 0 | 0 | 1 | 0 | 0 | 0 | EU650416.1 | Lima Jr and Scarpassa (2009) |
| Hap_18 | 0 | 0 | 0 | 0 | 57 | 0 | 0 | 0 | 0 | AF334865.1 | Gorrochotegui-Escalante et al. (2002) |
| Hap_19 | 0 | 3 | 0 | 0 | 26 | 0 | 0 | 0 | 0 | DQ176831.2 | Bracco et al. (2007) |
| Hap_20 | 0 | 2 | 0 | 1 | 0 | 0 | 0 | 0 | 0 | EU446272.1 | Burugu et al. unpub |
| Hap_21 | 0 | 0 | 0 | 0 | 12 | 0 | 0 | 0 | 0 | AF334848.1 | Gorrochotegui-Escalante et al. (2002) |
| Hap_22 | 0 | 0 | 0 | 0 | 17 | 0 | 0 | 0 | 0 | AF334849.1 | Gorrochotegui-Escalante et al. (2002) |
| Hap_23 | 0 | 0 | 0 | 0 | 18 | 0 | 0 | 0 | 0 | AF334857.1 | Gorrochotegui-Escalante et al. (2002) |
| Hap_24 | 0 | 0 | 0 | 0 | 3 | 0 | 0 | 0 | 0 | AF334861.1 | Gorrochotegui-Escalante et al. (2002) |
| Hap_25 | 0 | 0 | 0 | 0 | 4 | 0 | 0 | 0 | 0 | AF334847.1 | Gorrochotegui-Escalante et al. (2002) |
| Hap_26 | 1 | 0 | 0 | 0 | 0 | 0 | 0 | 1 | 0 | JQ303017 | This study |
| Hap_27 | 0 | 0 | 0 | 0 | 9 | 0 | 0 | 0 | 0 | AF334862.1 | Gorrochotegui-Escalante et al. (2002) |
| Hap_28 | 1 | 0 | 0 | 0 | 0 | 1 | 0 | 0 | 0 | EU650417.1 | Lima Jr and Scarpassa (2009) |
| Hap_29 | 0 | 1 | 0 | 0 | 0 | 0 | 0 | 0 | 0 | JQ303018 | This study |
| Hap_30 | 0 | 1 | 0 | 0 | 0 | 0 | 0 | 0 | 0 | AY906840.1 | Paduan and Ribolla (2008) |
| *Total* | 74 | 77 | 51 | 626 | 1983 | 27 | 14 | 11 | 22 |  |  |

BrAM: Brazilian Amazon; SEBr: Southeastern Brazil; PE: Peru; VZ: Venezuela; M-NA: Mexico-North America; MAO: Manaus; BEL: Belém; BV: Boa Vista; PV-RB: Rio Branco-Porto Velho

**Genetic diversity results**

*Continental scale*

Relative to sample size, Venezuela (*N* = 626) and Mexico-North America (*N* = 1983) had a similar number of haplotypes (0.011 haplotypes/sample), while the Brazilian Amazon (*N* = 74) and Southeastern Brazil (*N =* 77) had a higher number (~0.15 haplotypes/sample), and Peru (*N* = 51) had an intermediate value (0.04 haplotypes/sample). We observed a total of 22 segregating sites (*S*) across the whole sample (~7% of the 322 bases analyzed); within populations, *S* varied from 11 to 21 (Table S2).

Nucleotide diversity (*π*) values indicate a mean 2% sequence divergence across all samples, with differences ranging from 1.2 to 2% within individual populations (Table S2). In Peru and the Brazilian Amazon, *π* was larger than expected by the neutral coalescent (*p* < 0.025 using an empirical distribution of *π* from 1000 genealogies based on *S* values generated with the neutral coalescent simulation capability of DnaSP).

*Amazon scale*

Belém was the most variable population, with 0.50 haplotypes/sample, while Manaus and Rio Branco-Porto Velho had ~0.20 haplotypes/sample, which is similar to what was observed in Boa Vista (0.27 haplotypes/sample). We identified 15 segregating sites, with *S* varying from 6 to 13 across sampling sites.

Within-population nucleotide diversity was generally low, with mean pairwise differences ranging from 1.2 to 2.0 (*π* = 0.004 – 0.006; Table S2). The exception was Belém, with values closer to those observed at the continental scale (mean number of base differences per sequence = 5.8, *π* = 0.018; Table 1). Rio Branco-Porto Velho was the only population with a *π* value significantly smaller than expected by the neutral coalescent (*p* < 0.025 based on an empirical distribution of *π* values generated as described in the previous paragraph).

Table S2. Summary statistics for 2183 322bp ND4 mitochondrial sequences across two spatial scales in the Americas

| **Scale** | **Population** | **Molecular diversity indices** | | | | |
| --- | --- | --- | --- | --- | --- | --- |
|  |  | *N* | *h* | *S* | *Hd* ± *se^a^* | *π* ± *se^a^* |
| Continent | Mexico-North America | 1983 | 22 | 21 | 0.89 ± 0.003 | 0.0202 ± 8.7x10^-5^ |
|  | Venezuela | 626 | 7 | 13 | 0.65 ± 0.016 | 0.012 ± 0.0005 |
|  | Peru | 51 | 2 | 11 | 0.42 ± 0.054 | 0.014 ± 0.0018 |
|  | Brazilian Amazon | 74 | 12 | 15 | 0.76 ± 0.035 | 0.017 ± 0.0007 |
|  | Southeastern Brazil | 77 | 11 | 14 | 0.61 ± 0.061 | 0.013 ± 0.0016 |
|  | *Total* | *2811* | *30* | *22* | *0.90 ± 0.175* | *0.020 ± 0.0032* |
| Amazon | BoaVista | 11 | 4 | 6 | 0.60 ±0.154 | 0.005 ± 0.0016 |
|  | Manaus | 27 | 6 | 10 | 0.34± 0.117 | 0.006 ± 0.0026 |
|  | Belém | 14 | 7 | 13 | 0.81 ± 0.094 | 0.018 ± 0.0027 |
|  | Rio Branco-Porto Velho | 22 | 4 | 12 | 0.45 ± 0.115 | 0.004 ± 0.0026 |
|  | *Total* | *74* | *12* | *15* | *0.76 ± 0.203* | *0.017 ±0.0076* |

^a^ se: standard error calculated as described in equation 10.7 by Nei (1987)

**Parameter values for the F84 nucleotide substitution model used in Migrate-N runs**

Table S3. Maximum likelihood estimates of the parameters of the F84+Γ for each of the examined datasets obtained using PAUP*

| **Parameter** | **Amazon (*N* = 74)** | **Continent (*N* = 2811)** |
| --- | --- | --- |
| Frequency A (*π_A_*) | 0.280 | 0.280 |
| Frequency C (*π_C_*) | 0.080 | 0.080 |
| Frequency G (*π_G_*) | 0.200 | 0.200 |
| Frequency T (*π_T_*) | 0.440 | 0.440 |
| Transition/Transversion Ratio | 8.080 | 3.744 |
| Shape parameter of the Gamma distribution (α) | 0.031 | 0.004 |

**Posterior density**

Figure S1. Posterior density for all 15 parameters of the stepping-stone 1 model at the continental scale. Population labels: 1. Amazon; 2. Southeastern Brazil; 3. Peru; 4. Venezuela; and 5. Mexico-North America

See file supp.fig1.continent.ps

Figure S2. Posterior density for all 16 parameters of the full-migration model at the Amazon scale. Population labels: 1. Manaus; 2. Belém; 3. Boa Vista; and 4. Rio Branco-Porto Velho.

See File supp.fig2.amazon.ps

**Literature cited**

Bracco, J. E., M. L. Capurro, R. Lourenco-de-Oliveira, and M. A. M Sallum. 2007. Genetic variability of *Aedes Aegypti* in the americas using a mitochondrial gene: evidence of multiple introductions. *Memórias do Instituto Oswaldo Cruz* 102: 573–580.

Gorrochotegui-Escalante, N., C. Gomez-Machorro, S. Lozano-Fuentes, I. Fernandez-Salas, M. D. Munoz, J. A. Farfan-Ale, J. Garcia-Rejon, B. J. Beaty, and W. C. Black IV. 2002. Breeding structure of *Aedes aegypti* populations in Mexico varies by region. *American Journal of Tropical Medicine and Hygiene* 66: 213–222.

Nei, M. 1987. *Molecular evolutionary genetics*. Columbia University Press, New York.

Lima, R. S., and V. M. Scarpassa. 2009. Evidence of two lineages of the dengue vector *Aedes aegypti* in the Brazilian Amazon, based on mitochondrial DNA ND4 gene sequences. *Genetics and Molecular Biology* 32: 414–422.

Paduan, K. S., and P. E. M. Ribolla. 2008.“Mitochondrial DNA polymorphism and heteroplasmy in populations of *Aedes aegypti* in Brazil. *Journal of Medical Entomology* 45: 59–67.
